# Tickle fetishism: pleasure beyond playfulness

**DOI:** 10.3389/fpsyg.2024.1342342

**Published:** 2024-04-03

**Authors:** Sarah Dagher, Shimpei Ishiyama

**Affiliations:** Institute of Pathophysiology, University Medical Center of the Johannes Gutenberg University Mainz, Mainz, Germany

**Keywords:** tickling, sexual behavior, dominance, submission, satisfaction

## Abstract

Tickling is commonly perceived as juvenile play associated with laughter. However, its potential connection to adult sexual behavior has largely remained unexplored. Our online survey, primarily distributed among individuals interested in tickle fetishism, explored tickling and its association with sexual behavior. Ticklishness types, tools, preferred body parts, and partner preferences, were examined. Results revealed diverse patterns of ticklishness changes over time and distinct body-part preferences for different types of tickling. Childhood experiences and exposure to tickling content in television were found to shape individuals’ affinity for tickle fetishism. A quarter of respondents reported experiencing orgasms exclusively from tickling, while around 88% expressed sexual satisfaction through tickling alone, indicating its sufficiency as a sexual stimulus among fetishists. Tickling desire decreased after orgasm, indicating an association between tickling and sexual activity. Moreover, ticklishness degree predicted preferences for being tickled rather than tickling others. Exploratory factor analysis identified three factors underlying tickling and sexual experiences: enjoyment and frequency of tickling during sexual activity; preference for intense sexual experiences; age of becoming sexually active. In conclusion, this study provides unique insights into tickling and its connections to sexual context, enhancing our understanding of diverse human sexual behavior and tickle fetishism as a distinct preference.

## 1 Introduction

Tickling has long been a subject of fascination for scholars and intellectuals throughout history, including Darwin, Freud, and Aristotle (Darwin, 1872; Cooper, 1922; Freud, 1938). This intriguing phenomenon holds significant importance in human interactions, often serving as a mean of fostering social cohesion and bonding (Ishijima and Negayama, 2017), emotional connection (Szameitat et al., 2009) and eliciting amusement (Provine, 2012). Two types of tickling have been identified: gargalesis and knismesis (Hall and Allin, 1897). Gargalesis involves vigorous and playful tickling, leading to robust laughter and uncontrollable reactions from the ticklee. In contrast, knismesis represents a milder and more subtle form of tickling, triggered by gentle touches or movements on the skin. The response to knismesis is generally characterized by a slight discomfort or tingling sensation rather than the intense laughter observed in gargalesis. Tickling may reflect power dynamics within relationships, influencing the expression and negotiation of social roles and boundaries. Other scholars have described tickling to be the building block to humor development in infants (Fridlund and Loftis, 1990).

Throughout history, tickling has left imprints in ancient literature, artistic representations, and cultural customs. Philosophers like Aristotle contemplated its nature, particularly the elicitation of laughter through ticklish stimuli (Cooper, 1922). However, the perception of tickling has exhibited variability across diverse societies and historical epochs reflecting the diversity of human experiences and cultural interpretations. While some cultures embraced it as a form of entertainment, others viewed it with suspicion or employed it as a form of punishment or torture (Schreiber, 2001; Robinson, 2006; Beard, 2014). Tickling has found applications in laughter stress relief studies (Akimbekov and Razzaque, 2021), extending to animal models (Burgdorf and Panksepp, 2001; LaFollette et al., 2017). Tickling has also been explored as a non-threatening intervention in certain forms of therapy (Greene and Hoats, 1971), prompting the development of wearable tickling instruments (Elvitigala et al., 2022).

Despite the common occurrence of tickling in human societies, it remains a notably underrepresented topic in touch studies. This gap in research is highlighted by a recent large-scale meta-analysis of studies on touch interventions and well-being, which found no instances of tickling in over 200 studies (Packheiser et al., 2023). Nevertheless, there are some notable studies of tickling in the field of psychology often exploring the neural and psychological mechanisms underlying tickling sensations, as well as its relationship to laughter, playfulness, and bonding (Hall and Allin, 1897; Harris and Christenfeld, 1999; Verónica Juárez-Ramos et al., 2014; Ishijima and Negayama, 2017; Elise Wattendorf et al., 2019; Hammond et al., 2019). Neuroscientists have explored the processing of touch within the nervous system, as well as the brain regions and nerve pathways implicated in the tickling response (Zotterman, 1939; Ruggieri and Milizia, 1983; Ruggieri et al., 1985; Carlsson et al., 2000; Wattendorf et al., 2013, 2016; Ishiyama and Brecht, 2016; Leavens and Bard, 2016; Elise Wattendorf et al., 2019; Ishiyama et al., 2019). In the field of physiology, researchers have examined the physiological responses to tickling and touch (Drescher et al., 1985; Weiss, 1992), such as changes in heart rate (Drescher et al., 1980), hormones (Hori et al., 2013), facial expressions (Davila-Ross et al., 2015; Proelss et al., 2022) and muscle tone (Ruggieri et al., 1983). Some studies have explored the evolutionary significance of tickling and its potential adaptive functions in human interactions (Leavens and Bard, 2016). The evolutionary perspective seeks to understand why humans and certain animals find tickling pleasurable and engaging.

While extensive research has focused on human sexual behavior including fantasies (Lehmiller, 2018), the surprising involvement of tickling in sexual practices remains relatively unknown to many individuals, making the exploration of sexual tickling largely neglected in scientific research. This aspect of tickling is primarily recognized within a small niche community. However, with the rise of social media, there has been a recent increase in visibility, allowing these individuals to gain more recognition among a broader audience especially after the breakout of the documentary “Tickled” in 2016, directed by David Ferrier and Dylan Reeve. It has been argued that playful childhood tickling develops into adult sex play (Provine, 2012). In his questionnaire data, Provine collected answers endorsing this hypothesis. For instance, he showed that tickling was a popular form of sexual foreplay among both male and female respondents. Knismolagnia/Knismophilia/Titillagnia, also known as tickling fetishism, refers to individuals who experience sexual arousal or derive sexual pleasure from tickling others or being tickled (Aggrawal, 2009). Various types of fetishes have been investigated, but notable omissions included the assessment of tickle fetishism (Scorolli et al., 2007).

In the context of tickle fetishism, BDSM (Bondage, Discipline, Dominance, Submission, Sadism, Masochism) represents a spectrum of erotic practices that involve power dynamics and sensory play. The roles of tickler and ticklee in tickle fetishism can be analogized to the dominant and submissive roles in BDSM, where the act of tickling can be both a form of sensory play and an expression of power dynamics. Furthermore, vigorous tickling can incorporate elements of sadomasochism, as it often straddles the line between pleasure and discomfort. This duality of tickling, uniquely poised between enjoyment and unease, distinguishes it as a distinctive sensory experience within BDSM contexts. This relationship underscores the intricate connection between tickle fetishism and BDSM, contributing to the complexity of tickling experiences within the broader framework of sexual behavior. Despite valuable insights into the biology of BDSM, including brain activation and hormonal changes (Wuyts and Morrens, 2021), existing literature lacks a comprehensive understanding on tickle fetishism. Tickling fetishism shows an overlap with other fetishes, including foot fetishism (podophilia), suggesting potential connections between these various sexual preferences (Provine, 2012).

As an observational exploration of tickling and its intersections with sexual behavior, our primary objective was to systematically catalog and analyze existing practices and preferences. Given the scarcity of prior research on this distinct subject, our study focuses on the collection of valuable data which can contribute to the understanding of a puzzling and notable gap in the literature and the possible rise of theories governing such behaviors. Tickling holds significance in intimate relationships by functioning as a unique element that extends beyond the physical. It serves as a mechanism to foster closeness and intimacy between partners, operating as a bridge that goes beyond conventional forms of touch. In the sexual context, tickling introduces a distinctive dimension of playfulness, contributing to the emotional intricacies of intimate encounters. This playfulness adds diversity to the emotional repertoire of sexual experiences, facilitating shared laughter, moments of vulnerability, and the exploration of sensory stimuli. Thus, the scientific importance of tickling in intimacy lies in its capacity to bridge the tactile aspect with emotional and sensory dimensions, enriching the overall experience of human sexuality. We aim to explore tickle enjoyment in intimate contexts, its connection to sexual practices, and its potential impact on overall sexual satisfaction. Utilizing an online survey and a systematic methodology, we introduce the first comprehensive and detailed analysis of the unique association between tickling and human sexuality.

## 2 Materials and methods

### 2.1 Participants

The study recruited participants through an online questionnaire of 43 questions posted on Twitter (currently known as X) by the authors. Among several tickle fetish influencers contacted unsolicited on Twitter, two Japanese- and three English-speaking influencers, with a combined followers of 27,956, responded and agreed to cooperate with our study. We specifically requested these influencers to retweet the survey link and, if possible, to pin the tweet to their profiles. Two Japanese influencers complied by pinning the tweet for 3 weeks, after which we requested them to unpin it. The survey was available in both English and Japanese languages and remained open for responses over a duration of 108 days. A total of 719 individuals agreed with participation in the survey with 193 and 526 responses for English and Japanese versions, respectively. We did not offer any incentives for completing the questionnaire, as the study aimed to preserve participant anonymity and authenticity of responses. Thus, uniqueness of respondents was not confirmed. Nevertheless, the survey’s completion time was approximately 10 min, which we believe further minimized the likelihood of multiple submissions by the same individual. Participants were provided with a clear and concise description of the study’s purpose and informed consent was obtained prior to participation. It was confirmed by local authorities that formal ethical approval was not required because no personally identifiable information was collected, ensuring participant privacy and confidentiality.

### 2.2 Survey design

**Figure 1:**
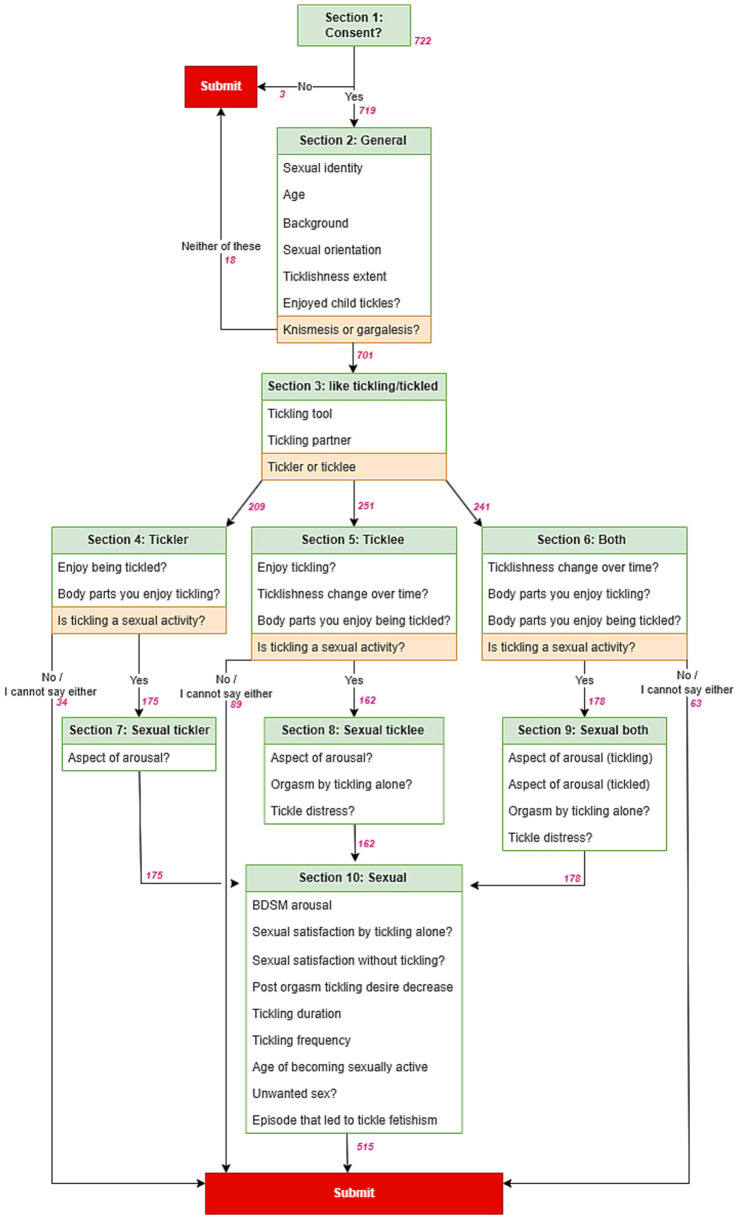
Structure of questionnaire. The questionnaire consisted of 10 sections. Participants were directed to subsequent sections based on their answers to specific questions highlighted in orange. Red numbers indicate number of respondents for each section. For exact questions, see Supplementary Table S1.

The questionnaire was made on Google Forms and included both multiple-choice and free-text formats to measure various aspects related to tickling. It covered topics such as tickling preferences, ticklishness extent, tickling tools and partners, and the potential connection between tickling and sexuality. The study adopted a dual approach, starting with a comprehensive examination of general aspects of tickling to establish a foundational understanding of ticklishness. Subsequently, a focused look into the sexual dimensions of tickling was pursued. This dual-phase strategy was designed to discern potential interrelations and influences between general ticklish experiences and specific manifestations in sexual behaviors. We provide a detailed description of questions and targeted participants of each section shown in the flowchart (Figure 1; Supplementary Table S1). For multiple-choice questions, participants were presented with predefined response options or the opportunity to provide their own answers in free-text format. Questions requested to select either single answers or multiple responses that applied to their experiences. The answer types are categorized in Supplementary Table S1 “Answer type” column as follows: “single” - indicating the selection of only one option; “multiple” - enabling participants to choose all applicable responses; “+ free text” - offering an “Other” option where participants could provide their own answers in their own words; “free text” - allowing unrestricted input without the constraints of multiple-choice options.

### 2.3 Data analysis

The Japanese version of the survey was translated into English by the author, who is a native speaker, after which the data from both versions were combined into a single dataset. Participants who did not provide consent were excluded. Subsequently, we merged responses for same questions asked in different sections among ticklers and individuals who identified as both ticklers and ticklees, as well as between ticklees and those identifying as both ticklers and ticklees (see Figure 1). The merged responses specifically focused on aspects such as body parts, the perception of tickling as a sexual activity, changes in ticklishness, arousal associated with tickling, experiences of orgasm, and experiences of distress. All analyses were performed using Exploratory v6.10 software (Exploratory, Inc., United States).^1^ Descriptive statistics were calculated to summarize the participants’ demographic characteristics, tickling preferences, changes in ticklishness, childhood experiences, and the sexual aspects of tickling. For multiple-choice questions with “Other” option and free-text comments, responses were manually categorized by the authors (see Supplementary Table S1, “Answer type”). Gender-based analysis (Supplementary Table S2) focused on male and female respondents, and did not include other genders, such as non-binary, questioning, and transgenders, due to the small sample size.

For the Chi-square analysis investigating the relationship between childhood enjoyment of tickling and ticklers’ enjoyment of being tickled, responses regarding childhood enjoyment were transformed into logical data by excluding “I’m not sure/I do not remember.” Similarly, in the chi-square analysis exploring the preference to tickle someone or to be tickled, and ticklishness extent, participants who selected “Both equally” from the question “Do you prefer to tickle or to be tickled?” were excluded from the analysis.

The correlation analysis between ordinal data was conducted using the Spearman method, with the data converted to numerical type. For example, the following numerical conversions were applied: 1: “Completely unticklish”; 2: “Somewhat unticklish”; 3: “Neither ticklish nor unticklish”; 4: “Somewhat ticklish”; 5: “Extremely ticklish.” In instances where questions included both ordinal (e.g., “I experience orgasm every time with tickling alone”) and non-ordinal answers (e.g., “I do not know”), the non-ordinal answers were excluded from the correlation analysis. Exploratory factor analysis (EFA) (Tavakol and Wetzel, 2020) was performed among ordinal data, similar to the correlation analysis. Number of factors was defined by Exploratory software according to scree plot.

## 3 Results

### 3.1 Survey design

To study the relationship between tickling and sexual behavior, we conducted an online survey. The survey was distributed on X (formerly known as Twitter), primarily targeting individuals with an interest in tickle fetishism. A total of 719 participants agreed to take part in the online questionnaire (Figure 1; Supplementary Table S1). The survey design followed a sequential approach, starting with a demographic, general questions about tickling, and the sexual dimension of tickling experiences. The questionnaire adopted a progressively selective approach targeting individuals based on their answers to questions about their enjoyment of tickling sensations, preference for tickling (“tickler”) or being tickled (“ticklee”), and the consideration of tickling as a sexual activity (Figure 1, orange highlights).

### 3.2 Participants characteristics

Participants’ demographics including age, gender, background, and sexuality are presented in Table 1. Statistics revealed a predominance of males (74.3%), followed by females (20.7%). The majority identified as East Asian (73.3%), while North Americans constituted 13.8% and Europeans 8.3%. Other backgrounds were represented by <1% of the participants. In terms of sexuality, most identified as heterosexual (79.7%), with smaller proportions as bisexual (10.8%) and asexual (2.8%) among others. Age distribution showed the majority in the 20–29 age range (56.3%), with participants aged 18–19 accounting for 14.2% and those aged 30–39 comprising 22.5% of the sample. The remaining age ranges represented <5% of the participants combined. A substantial number of participants expressed their appreciation for our study in the free comments section of the survey, indicating a genuine interest among the respondents in contributing to a better scientific understanding of tickle fetishism.

**Table 1:** Summary of demographics.

Gender (*n* = 719)	Participants (%)
Male	74.3
Female	20.7
Non-binary	2.4
Prefer not to say	2.1
Male (questioning)	0.3
Transgender male-to-female	0.1
Transgender female-to-male	0.1
Background (*n* = 719)	
East Asian	73.3
North American	13.8
European	8.3
Latin American	3.1
South East Asian	0.4
Australian	0.3
Middle Eastern	0.3
African	0.1
Haitian	0.1
Hispanic	0.1
NZ European	0.1
Sexuality (*n* = 719)	
Heterosexual	79.7
Bisexual	10.8
Asexual	2.8
Prefer not to say	2.8
Homosexual	2.6
Queer/pansexual	1.1
Demisexual	0.1
Age (*n* = 719)	
Prefer not to say	1.4
18–19	14.2
20–29	56.3
30–39	22.5
40–49	3.8
50–59	1.3
60–69	0.6

### 3.3 General tickling characteristics

**Figure 2:**
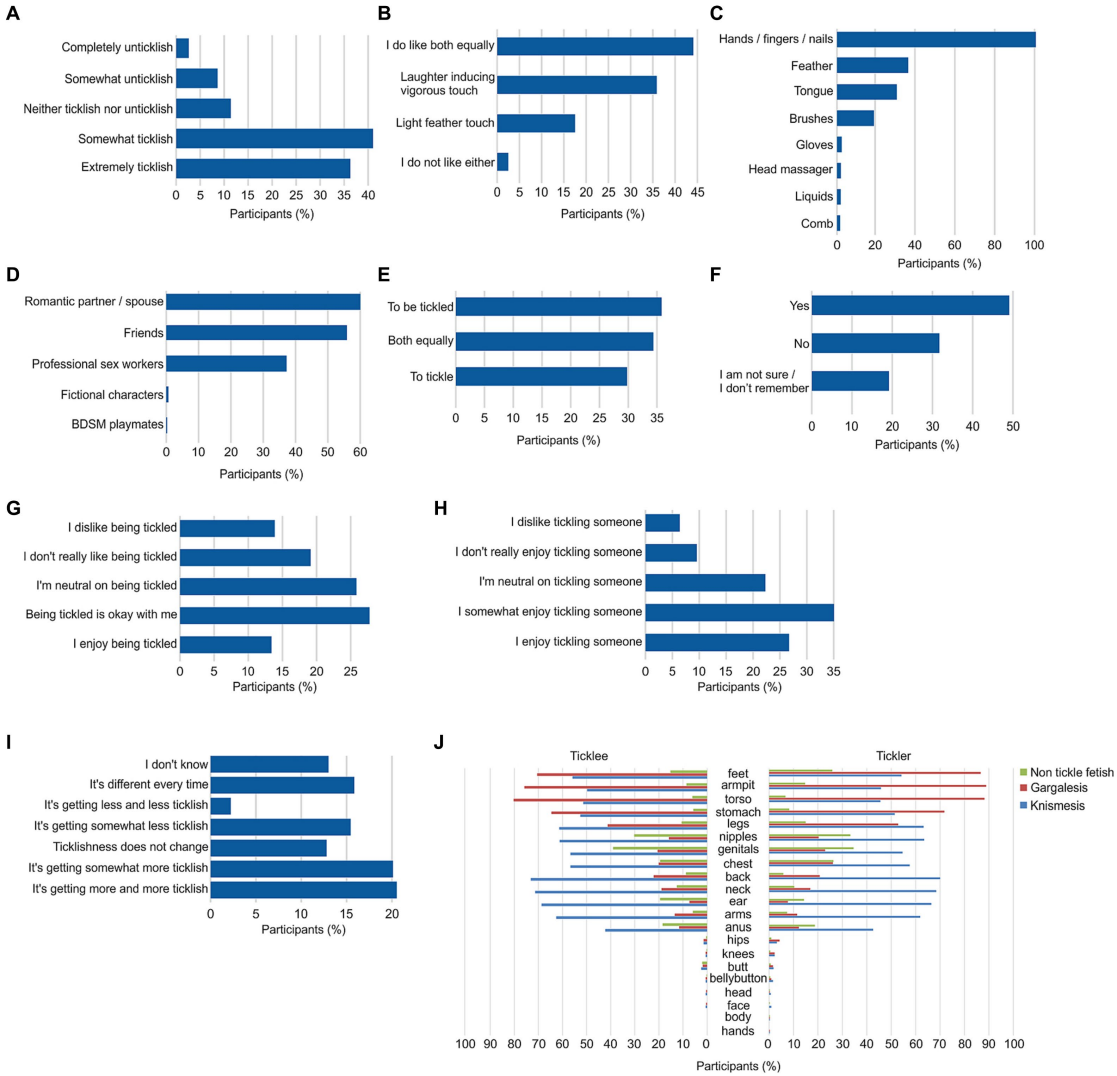
General tickling characteristics of participants. **(A)** Ticklishness extent (*n* = 719). **(B)** Type of tickle sensation preferred (*n* = 719). **(C)** Top 8 tools used for tickling (*n* = 701). **(D)** Top 5 tickling partners (*n* = 701). **(E)** Participants’ preference to tickle or be tickled (*n* = 701). **(F)** Enjoyment of tickling during childhood (*n* = 719). **(G)** Enjoyment of being tickled for ticklers (*n* = 209). **(H)** Enjoyment of tickling for ticklees (*n* = 251). **(I)** Ticklishness change over time (*n* = 492). **(J)** Ticklish body parts and fetish preferences (tickling *n* = 450, being tickled *n* = 492). Percentages in **C,D** reflect respondents’ multiple-choice selections which may result in a total exceeding 100%.

Overall, 77.4% of the participants reported being ticklish (Figure 2A, “Somewhat ticklish” and “Extremely ticklish”), with 44.1% expressing enjoyment for both light and heavy tickling (Figure 2B). Gender-based analysis revealed that females tend to be more ticklish, and prefer light tickling (Supplementary Table S2). Notably, a vast majority of participants (99.7%) used their hands/fingers/nails as the primary tool for tickling, while other prevalent tools included feathers, tongues, and brushes (Figure 2C). Additionally, participants selecting “other” and providing their own words reported using other tools such as gloves, massagers, liquids (e.g., lubricants/oils), and combs in their tickling practices. Tickling activities were predominantly performed with romantic partners or spouses (60.1%) and friends (55.9%) (Figure 2D). Participants who chose the “other” option reported engaging in tickling with fictional characters and BDSM playmates. Regarding preferences for tickling or being tickled, the distribution showed a relatively balanced representation, with 35.8% identifying as ticklees, 29.8% as ticklers, and 34.4% expressing enjoyment for both roles (Figure 2E), whereas gender-based analysis showed that 76% of female respondents preferred to be tickled (Supplementary Table S2). Notably, approximately one-third of the participants (31.7%) reported not enjoying being tickled during childhood (Figure 2F). Among ticklers, 41.2% had no issue with being tickled (Figure 2G, “Being tickled is okay with me” and “I enjoy being tickled”), while 33.0% expressed dislike for being tickled (“I do not really like being tickled” and “I dislike being tickled”). In addition, more than half of ticklees (62.8%) reported enjoying tickling someone (Figure 2H, “I somewhat enjoy tickling someone” and “I enjoy tickling someone”). To investigate potential differences in the enjoyment of the opposite role between ticklers and ticklees, we employed a t-test on the numerical representations of responses to the respective questions. Notably, a significant difference was observed between ticklers and ticklees in terms of their enjoyment of the opposite role (*p* < 0.001; t-stat = −5.2; DF = 458; Cohen’s D = 0.49; t-test). Specifically, ticklees were found to derive higher enjoyment from tickling someone compared to ticklers’ enjoyment in being tickled. Furthermore, the change in ticklishness degree over the course of continuous tickling exhibited considerable variability among ticklees (Figure 2I). An increase in ticklishness was reported by 40.6% (“getting somewhat more ticklish” and “getting more and more ticklish”), while 15.8% noted that the sensation varied each time, indicating that this subjective feeling might be influenced by various factors. Notably, gender-based analysis showed females reported a predominant increase of ticklishness (Supplementary Table S2). When exploring preferred body parts for tickling, our study revealed a consistent body map pattern between tickler and ticklees (including those who identify as “both,” Figure 2J). Gargalesis, a more intense form of tickling, was mainly focused on the feet, armpits, torso, and stomach. In contrast, light tickling or knismesis uniformly extended over the entire body including the back, neck, ears, genitals, and arms, among others. As expected, other forms of non-tickle fetishisms were predominantly associated with the nipples and genitals. Hips, knees, butt, bellybutton, head, face, body, and hands were body parts described by the participants’ own words.

### 3.4 Sexual aspect of tickling

**Figure 3:**
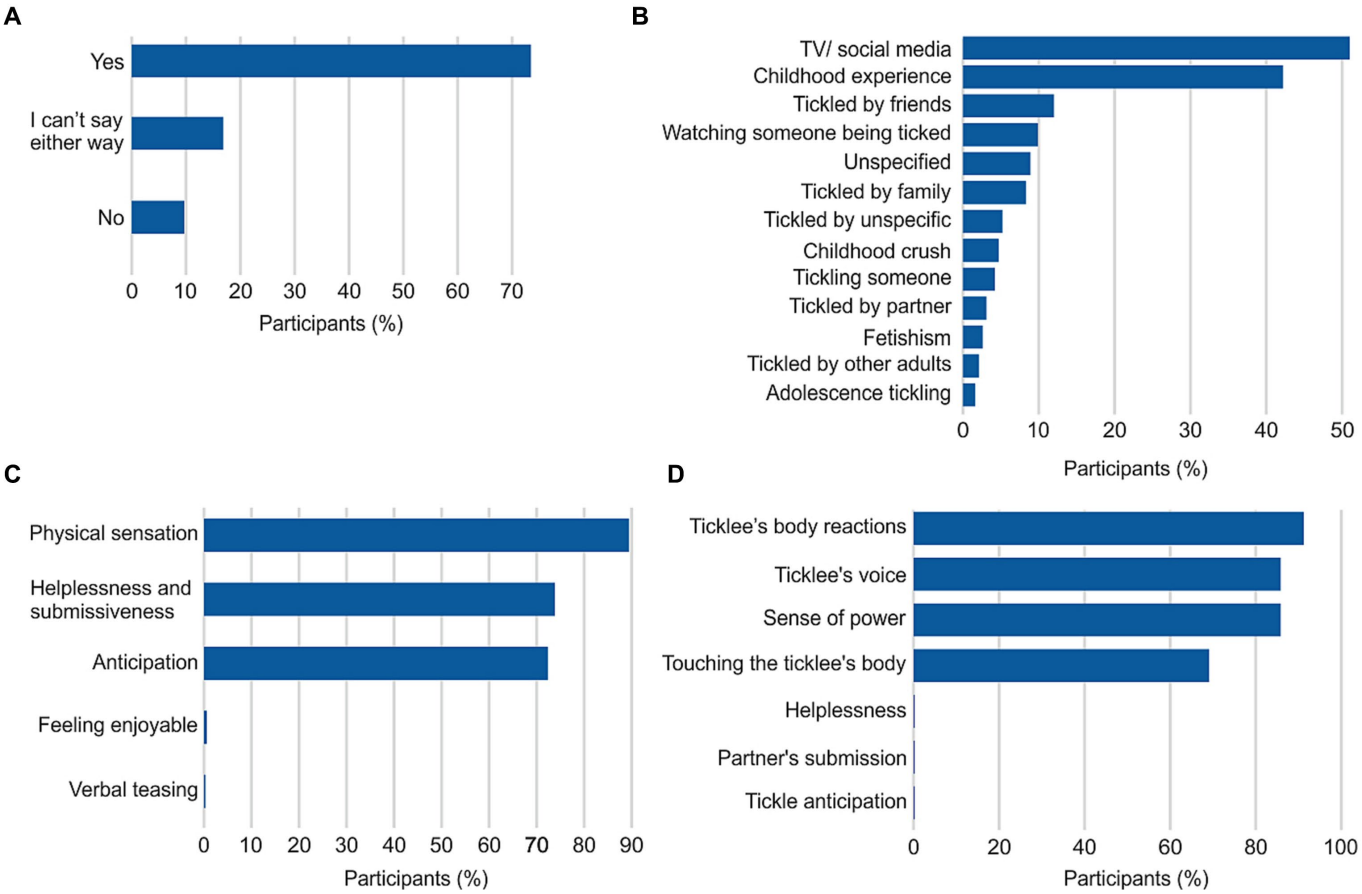
Sexual aspect of tickling. **(A)** Tickling as a sexual activity (*n* = 701). **(B)** Episodes leading to tickle fetishism (*n* = 515). **(C)** Factors of tickle sexual arousal for being tickled (*n* = 340). **(D)** Same as **(C)** but for tickling others (*n* = 353). Percentages in **B** reflect respondents’ multiple-choice selections which may result in a total exceeding 100%.

Given the survey’s primary distribution among individuals with an interest in tickle fetishism, more than two-thirds of participants reported that tickling holds a sexual connotation for them (Figure 3A). Intriguingly, exposure to tickling scenes in television (e.g., Teenage Mutant Ninja Turtles, Popeye, Kiki’s Delivery Service, Lupin the Third) and social media constituted a significant contributing factor (51%) to the development of tickle fetishism, as reported by participants (Figure 3B). Childhood experiences of being tickled were also commonly cited as a starting point for the development of tickle fetishism (42.2%). To gain insights into the arousing factors of tickling, ticklees predominantly reported the physical sensation of being tickled and their bodily response as the primary reasons (89.4%) for arousal (Figure 3C). Additionally, feelings of helplessness and submissiveness (73.8%), as well as the anticipation of being tickled (72.4%), were identified as important arousing elements. Other factors described were feeling enjoyable and tickler’s verbal teasing. On the other hand, arousal in ticklers appeared to arise from a more diverse range of factors (Figure 3D). The main source of arousal was visually observing the ticklee’s body reactions (91.2%), followed by the sound of the ticklee’s voice (85.8%) and the sense of power gained through tickling (85.8%). Tactile stimulation was also reported as an arousal source by 69.1% of participants. Other factors described were the ticklees’ helplessness and submission, as well as their tickle anticipation.

### 3.5 Sexual preferences and tickle practices

In the exploration of tickle fetishism and its associations with sexual behavior, several notable findings emerged from the data (Table 2). Around a quarter of participants (25.8%) reported never engaging in sexual activity, while the age range of 20–29 years represented the highest proportion (32.6%) for the initiation of sexual activity. Additionally, approximately 6.6% of participants reported experiencing unwanted sexual experiences during their childhood. A noteworthy 55.8% (“to some extent” and “I do very much”) of participants felt sexually aroused by BDSM acts other than tickling. Notably 23.9% of participants have experienced orgasm with tickling alone while 58.5% reported never experiencing it. Interestingly, 52.2% (“desire decreases somewhat” and “desire decreases significantly”) of participants reported a decrease in desire to tickle or be tickled after orgasm. Furthermore, 45.3% (“somewhat satisfied” and “very much satisfied”) of participants reported being satisfied with sexual activity not involving tickling. A striking 88.1% (“somewhat satisfied” and “fully satisfied”) expressed sexual satisfaction solely from tickling. Some participants (37.6%, “I experience some distress” and “I experience significant distress”) reported experiencing distress when tickled. In terms of frequency and duration of tickling as a sexual activity, the majority (33.8%) engaged in tickling for 10–29 min continuously, and 20.8% reported performing tickling every time in their sexual activity.

**Table 2:** Sexual preferences and tickle practices.

Age of sexually active (*n* = 515)	Participants (%)
Prefer not to say	1.4
I have never engaged in sexual activity	25.8
Below 15	6.4
15–19	31.5
20–29	32.6
30–39	2.1
40–49	0.2
Unwanted sex (*n* = 515)	
I do not remember	5.0
No	86.4
Prefer not to answer	1.9
Yes	6.6
Arousal by BDSM (*n* = 515)	
I have no experience of BDSM/I do not know	13.2
Not at all	8.9
Not very much	17.3
I am neutral/I cannot say either way	4.9
Yes, to some extent	34.8
Yes, I do very much	21.0
Orgasm (*n* = 515)	
I do not know	17.6
I have never experienced orgasm with tickling alone	58.5
I have experienced orgasm a few times with tickling alone	10.0
I experience orgasm sometimes with tickling alone	6.5
I experience orgasm every time with tickling alone	7.4
Desire decrease (*n* = 515)	
I have never experienced orgasm	6.0
No, desire does not decrease at all	10.5
No, desire does not decrease very much	17.1
I cannot say either way	14.2
Yes, desire decreases somewhat	40.4
Yes, desire decreases significantly	11.8
Non tickle satisfaction (*n* = 515)	
I have no experience of sexual activity	19.4
I have no experience of sexual activity that does not involve tickling	2.5
Not satisfied at all	5.8
Somewhat not satisfied	16.9
I cannot say either way	10.1
Somewhat satisfied	33.8
Very much satisfied	11.5
Tickle satisfaction (*n* = 515)	
Not satisfied at all	1.6
Somewhat not satisfied	5.8
I cannot say either way	4.5
Somewhat satisfied	55.1
Fully satisfied	33.0
Distress (*n* = 515)	
I experience no distress	19.7
I experience little distress	28.2
I cannot say either way	14.4
I experience some distress	29.7
I experience significant distress	7.9
Tickle duration (*n* = 515)	
Less than 10 min	25.8
10–29 min	33.8
30–59 min	17.7
Longer than 60 min	22.7
Tickle frequency (*n* = 515)	
I have no experience of sexual activity	23.3
I have never engaged in tickling in my sexual activity	7.6
Rarely	10.9
Occasionally	16.7
Quite often	20.8
Every time	20.8

### 3.6 Predictors of tickle enjoyment in adulthood

Participants’ tickle preferences were influenced by their childhood experiences (Table 3). A majority (70.1%) of ticklees enjoyed childhood tickling compared to only 20.6% of ticklers (*p* < 0.001; Chi-square = 115.61; DF = 2; Cohen’s W = 0.41). In contrast, participants engaging in both roles as ticklers and ticklees showed more balanced experience for childhood tickling. On the other hand, results of the chi-square tests examining the association between childhood tickling enjoyment and tickle enjoyment in adulthood indicated significant findings (*p* < 0.001; Chi-square = 33.13; DF = 4; Cohen’s W = 0.40). Consistent with the previous results, individuals who reported enjoying childhood tickling, a considerable proportion (69.8%, “Being tickled is okay with me” and “I enjoy being tickled”) expressed enjoyment in being tickled by someone in adulthood, while only a minor percentage (9.4%, “I do not really like being tickled” and “I dislike being tickled”) disliked being tickled. On the other hand, among those who disliked childhood tickles, the majority (39.2%, “I do not really like being tickled” and “I dislike being tickled”) also expressed a dislike for being tickled in adulthood. Similarly, the chi-square tests investigating the relationship between ticklishness extent and preference for tickling or being tickled in adulthood yielded significant results (*p* < 0.001; Chi-square = 61.29; DF = 4; Cohen’s W = 0.37). Participants who identified as unticklish were more inclined (20.1%, “Completely unticklish” and “Somewhat unticklish”) to prefer tickling others, while a smaller proportion (6.0%, “Completely unticklish” and “Somewhat unticklish”) preferred to be tickled. Conversely, those who considered themselves ticklish were more likely (85.7%, “Extremely ticklish” and “Somewhat ticklish”) to prefer to be tickled, with a lesser percentage (64.1%, “Extremely ticklish” and “Somewhat ticklish”) indicating a preference to tickle others.

**Table 3:** Predictors of tickle enjoyment in adulthood.

Tickle preference	Enjoy childhood tickles	Dislike childhood tickles	*p*-value
Both equally	55.2	44.8	< 0.001
To be tickled	70.1	29.9	
To tickle	20.6	79.4	
Enjoy being tickled	Enjoy childhood tickles	Dislike childhood tickles	*p*-value
I dislike being tickled	4.7	16.3	< 0.001
I do not really like being tickled	4.7	22.9	
I’m neutral on being tickled	20.9	27.1	
Being tickled is okay with me	32.6	26.5	
I enjoy being tickled	37.2	7.2	
Tickle extent	To be tickled	To tickle	*p*-value
Completely unticklish	0.8	5.3	< 0.001
Somewhat unticklish	5.2	14.8	
Neither ticklish nor unticklish	8.4	15.8	
Somewhat ticklish	35.1	45.9	
Extremely ticklish	50.6	18.2	

### 3.7 Quantitative analysis of tickle fetishism

**Figure 4:**
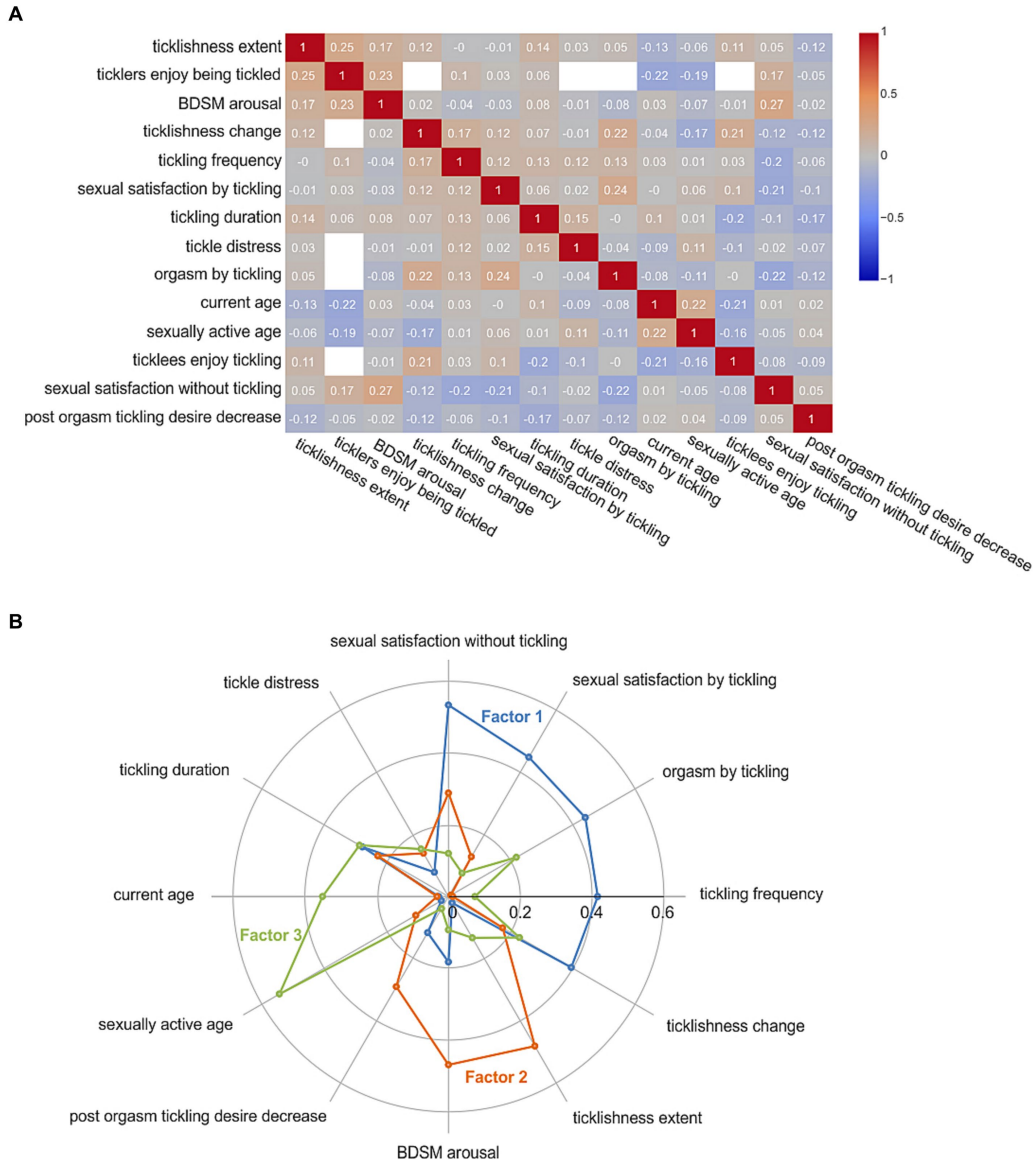
Quantitative analysis of tickle fetishism. **(A)** Correlation matrix of tickle fetishism parameters. For value of *p*s, see Supplementary Table S3. **(B)** Exploratory factor analysis radar chart of absolute variable loads. For more details, see Supplementary Table S4.

The correlation matrix (Figure 4A; Supplementary Table S3) revealed several significant associations between different variables related to tickling and sexual behavior. Notably, there was a positive correlation (*r* = 0.27, *p* < 0.001) between BDSM arousal and sexual satisfaction without tickling. Sexual satisfaction by tickling alone showed a negative correlation with sexual satisfaction without tickling (*r* = −0.21, *p* = 0.001). Furthermore, orgasm by tickling demonstrated a positive correlation with sexual satisfaction by tickling alone (*r* = 0.24, *p* = 0.003) and ticklishness change (*r* = 0.22, *p* = 0.006), but a negative correlation with sexual satisfaction without tickling (*r* = −0.22, *p* = 0.006). Additionally, ticklishness extent exhibited positive correlations with BDSM arousal (*r* = 0.17, *p* = 0.005), ticklers enjoying being tickled (*r* = 0.25, *p* = 0.007), and negative correlation with current age (*r* = −0.13, *p* = 0.036). Exploratory factor analysis (EFA) yielded 3 factors that explain underlying associations between variables (Figure 4B; Supplementary Table S4). Factor 1 demonstrated substantial loadings for variables related to sexual satisfaction in light of tickling as indicated by high coefficients for sexual satisfaction without tickling (−0.5), sexual satisfaction by tickling (0.4), tickling frequency (0.4), orgasm by tickling (0.4), and ticklishness change (0.4). Factor 2 exhibited high loadings of ticklishness extent (0.5) and BDSM arousal (0.5), whereas factor 3 was characterized by high loadings of sexually active age (0.5) and current age (0.4) suggesting that individuals who became sexually active at a younger age tend to be grouped together in this factor.

## 4 Discussion

This study investigated tickling and its associations with sexual behavior. A total of 719 participants were recruited for this research. However, it is crucial to acknowledge that the survey links were primarily disseminated within tickle fetishism communities on Twitter, predominantly comprising English and Japanese speakers. Because of this demographic recruitment bias, the generalizability of the findings to a broader population may be limited. Additionally, the utilization of anonymity in the survey prevented the verification of individual identities, thereby impeding follow-up clarifications or the collection of Supplementary information. Our quantitative analyses are exploratory in nature, and thus findings should be interpreted with an understanding of the potential for increased type I error rates.

We observed a predominance of responses from Japanese-speaking participants. This outcome is primarily attributed to the higher visibility of the Japanese tweet, facilitated by two Japanese influencers who kindly pinned the survey link on their Twitter accounts for 3 weeks. Thus, the Japanese tweet achieved 113,000 views and 47 retweets, in contrast to 26,800 views and 18 retweets for the English tweet. While the increased visibility in the Japanese-speaking community contributed to the higher number of Japanese respondents, it is important to consider other potential factors. These include the size of the Japanese tickle fetishist community on social media, the likelihood of Japanese individuals participating in such surveys, or a broader cultural interest in the subject within this community. Additionally, our survey showed a predominant male response across both language versions. The reason for this gender skew, possibly due to varying willingness to participate in such surveys, a greater social media activity among male fetishists, or cultural factors influencing gender expression in fetishism, remains unclear and presents an area for future research. These observations highlight the need for caution in generalizing our findings and suggest intriguing avenues for exploring cultural and gender variations in tickle fetishism.

The study uncovered intriguing patterns in tickling practices and preferences. A significant proportion of participants reported being ticklish and enjoying both gargalesis and knismesis. The wide scope and creativity in the practice of tickling was seen in the broad range of tools employed, including hands, feathers, liquids, and combs in addition to tickling partners. Prior research also acknowledged the relevance of fingertips and feathers in tickling experiences (Provine, 2012). Furthermore, while confirming the prevalence of tickling with romantic partners or friends as previously shown (Provine, 2012), the study identified a subset of participants with preference for BDSM- related playmates or fictional characters. This emphasizes the multifaceted nature of tickling experiences, extending beyond mere physical sensations to encompass emotional, psychological, and cognitive dimensions.

A clear separation between individuals’ preferred roles in tickling interactions, as either ticklers or ticklees, has been observed (Figure 2E), suggesting distinct psychological and emotional aspects at play in the tickling dynamic. For ticklers, initiating tickling may evoke feelings of power and control, contributing to emotional satisfaction. Conversely, ticklees may experience pleasure from the physical sensations induced by tickling and the associated vulnerability. This dynamic could potentially reflect different personality traits, such as dominance and submission tendencies, which can be intertwined with individuals’ preferences in their roles during tickling interactions. Furthermore, the distinction in enjoyment between ticklers and ticklees could also be influenced by cultural and personal factors. For instance, some individuals may find joy in taking on the role of the “tickler” as it aligns with traditional notions of being assertive and initiating intimate activities. On the other hand, those who enjoy being “ticklees” may experience pleasure from embracing a more submissive or receptive role. The significant difference observed between ticklers and ticklees in their enjoyment of the opposite role (Figures 2G,H) may be attributed to various factors. Firstly, being tickled may elicit more intense physiological responses compared to tickling somebody else. Secondly, this behavioral flexibility, i.e., the transition from being dominant to submissive might be more challenging for individuals who naturally adopt dominant roles. In contrast, individuals who are naturally submissive might find it comparatively easier to take on a dominant role. Interestingly, this concept has been shown in a previous study where dominant mice exhibit reduced resilience to social hierarchy changes, experiencing heightened susceptibility to stress compared to submissive mice (Larrieu et al., 2017). This observation suggests that the psychological dynamics and personal preferences play significant roles in shaping the enjoyment and comfort levels of ticklers and ticklees, and the process of shifting between these roles might differ based on an individual’s innate tendencies.

The observed distinct patterns in the preferred body parts targeted during gargalesis and knismesis pose intriguing inquiries concerning the underlying mechanisms and psychological aspects of tickling experiences. The non-tickle fetish body parts identified in our study, such as genitals and nipples, align with previously reported erogenous zones (Turnbull et al., 2014; Nummenmaa et al., 2016), whereas the body parts selected for gargalesis, including the feet, armpits, torso, and stomach, correspond less to these conventional erogenous zones, suggesting that the specificity of these body regions may be linked to their heightened sensitivity and vulnerability to tickling sensations. Prior research highlighted these areas as being particularly ticklish (Hall and Allin, 1897; Harris and Christenfeld, 1999; Provine, 2012), with some known to harbor a higher concentration of nerve endings (Corniani and Saal, 2020), rendering them more responsive to tactile stimuli. Consequently, the tickling response in these regions may be intensified, leading to heightened pleasure and amusement for both the tickler and the ticklee. Conversely, the more widespread and nonspecific distribution of knismesis body regions, such as the back, neck, ears, genitals, and arms as previously shown (Ruggieri and Milizia, 1983), suggests a preference for a subtler and generalized response to light touch stimulation. This broader involvement of various body regions could be associated with sensory receptors throughout the body, leading to a preference for a different perceptual experience compared to the localized intensity of gargalesis. This highlights the potential distinction of neurophysiological mechanisms involved in the processing of these stimuli.

Consistent with prior research on tickling (Selden, 2004; Provine, 2012), our study demonstrated that tickling can be indeed associated with sexual connotation. It is plausible to speculate that knismesis and sensual caress, even among individuals without tickle fetishism, could be interchangeable depending on the context or mental state. Previous research (Gazzola et al., 2012) supports the notion that the same physical stimulus can be perceived emotionally differently based on an individual’s current state of mind. In contrast, gargalesis, in the case of individuals without tickle fetishism, might be unlikely to serve as a significant sexual stimulus, highlighting the unique interplay between neurophysiological responses and psychological states. Sexual aspects of tickling were previously described in males who like being tickled, as components related to masturbation, sexual fantasies, erotism, arousal, and domination (Verónica Juárez-Ramos et al., 2014). Through exploratory factor analysis (factor 1, Figure 4B), we captured the fetish aspect of tickling reflecting individuals who experience tickling as a specific fetishistic interest or arousal trigger in their sexual lives. These individuals may find tickling as a powerful stimulant for sexual contentment.

A particularly intriguing finding was that a considerable number of participants reported experiencing orgasm solely from tickling, without any involvement of genital stimulation, which stands out in the context of existing literature. Studies that documented orgasm triggered by extragenital stimulation (Komisaruk and Whipple, 2011; Younis et al., 2016), and studies on ejaculation (Levin, 2005) do not specifically mention tickling as a stimulation method. Similarly, research focusing on tickling and sexual behaviors (Gutheil, 1947; Verónica Juárez-Ramos et al., 2014) do not establish a direct link between tickling and orgasm. The exception appears to be an early case study in a woman reaching orgasm through tickling (Ellis, 1913). This suggests that our findings may highlight an unexplored aspect of sexual response related to tickling. Possible speculation includes the activation of erogenous zones or nerve endings in the body as previously mentioned (Provine, 2012), leading to heightened arousal and pleasure. The psychosocial context in which tickling occurs may also play a role, as intimate and playful interactions with a partner during tickling could enhance the emotional and psychological aspects of sexual experiences. This combination of sensory and emotional factors may create a unique and potent sexual experience for individuals.

Our analysis revealed distinct gender-based differences in tickle fetishism (Supplementary Table S2). Females identified themselves more ticklish, preferred lighter tickling, and were more likely to be ticklees compared to males. They also reported greater enjoyment of childhood tickles and an increase in ticklishness during continuous tickling. These findings suggest notable gender variations in sensory perception, and preferences within tickle fetishism, highlighting the need for further research to understand these differences more deeply.

Furthermore, our exploratory factor analysis (factor 2, Figure 4B) revealed that individuals who reported higher levels of ticklishness and experienced greater arousal from BDSM-related activities tend to cluster together. One plausible explanation is the influence of overlapping neural or psychological processes. It is conceivable that individuals with heightened ticklishness possess an increased responsiveness to various sensory stimuli, including those involved in BDSM activities. Additionally, both ticklishness and BDSM arousal may be influenced by specific personality traits or psychological factors that predispose individuals to heightened sensations and arousal. Another speculation relates to the nature of BDSM activities, which often involve sensory play, power dynamics, and intense physical sensations. For individuals with greater ticklishness, the sensory aspects of BDSM may be particularly stimulating and arousing. Their heightened sensitivity to touch and sensation could enhance the overall experience of engaging in BDSM activities, thereby strengthening the association between ticklishness and BDSM arousal. It is essential to consider the psychological and emotional aspects of ticklishness and BDSM arousal. Both experiences could evoke feelings of vulnerability, excitement, and pleasure. Previous work described tickling as a form of sadomasochism (Selden, 2004; Provine, 2012). It is plausible that individuals who derive pleasure from the vulnerability and excitement of being tickled may also encounter similar emotional experiences in BDSM activities, leading to a positive correlation between these two factors.

An additional significant finding was the influence of television and media on the development of tickle fetishism. Many participants reported that exposure to tickling scenes in specific cartoons triggered their interest in tickling and contributed to the development of their fetishism. This underscores the potential influence of childhood media consumption on the establishment of adult preferences and desires. It raises questions about the importance of media content in shaping unconscious associations and sexual interests as previously explored (Brown, 2002), highlighting the need for more research on the effects of childhood experiences versus genetic predisposition on human sexuality and preferences.

In conclusion, this study makes a significant contribution to our understanding of tickling, moving beyond its traditional playful context to explore its associations with sexual behavior. As the first systematic investigation of its kind, this research provides detailed insights into various aspects of tickling experiences, shedding light on the complexities of tickle enjoyment and its potential role in sexual behavior. Moving forward, future studies should address the limitations of this survey, including expanding the sample size to encompass a broader demographic and exploring diverse cultural contexts. Experimental research focusing on the physiological and neural responses to tickling and sexual satisfaction could deepen our understanding of the underlying mechanisms. Specifically, the intriguing processes responsible for the dual interpretation of tickling as both playful and sexually relevant. For instance, extensive studies have explored hypothalamic circuits in the context of sexual behavior (Barfield, 1979; Iovino et al., 2019) and, to a certain extent, in play behavior (Lewis and Barton, 2006; Siviy, 2016). Given the convergence of hypothalamic functions, i.e., aggressive, sexual, feeding, and parental behaviors (Timper and Bruning, 2017; Haller, 2018; Orikasa, 2021), it raises the question of whether distinct sub-circuits within the hypothalamus could also contribute to the differentiation between tickling as a form of play and its involvement in more explicitly sexual contexts.

Additionally, exploring the role of personality traits and cultural factors in shaping tickling preferences and its sexual connotations would provide valuable insights into the intricate connections between human sexual behavior and individual differences.

## Data availability statement

The raw data for this article are not publicly available due to the informed consent agreement which did not explicitly include provisions for making the raw data publicly available. Further inquiries can be directed to the corresponding author.

## Ethics statement

The requirement of ethical approval was waived by local authorities because no personally identifiable information was collected, ensuring participant privacy and confidentiality. The studies were conducted in accordance with the local legislation and institutional requirements. The participants provided their written informed consent to participate in this study.

## Author contributions

SD: Visualization, Formal analysis, Writing – review & editing, Writing – original draft, Methodology, Investigation, Data curation, Conceptualization. SI: Supervision, Project administration, Funding acquisition, Writing – review & editing, Writing – original draft, Methodology, Investigation, Data curation, Conceptualization.

## Footnotes

^1^ https://exploratory.io
