# Supplementary material for: Tickle fetishism: pleasure beyond playfulness
Source: Front Psychol. 2024 Apr 3;15:1342342. doi: 10.3389/fpsyg.2024.1342342 (PMC11021705; doi:10.3389/fpsyg.2024.1342342)
Supplement: Supplementary file 1 [file Data_Sheet_1.PDF]

Supplementary Material

Table S1: List of questions

Section	Question as in flowchart	Full question	Answer type	Targeted participants
**1**	Consent?	By selecting "yes" below, you acknowledge that you have read the survey description above and agree to participate. Please select "no" if you do not wish to participate.	single	all
**2**	Sexual identity	Sexual identity	single + free text	all
**2**	Age	Age	single	all
**2**	Background	What is your cultural background?	single + free text	all
**2**	Sexual orientation	Sexual orientation	single + free text	all
**2**	Ticklishness extent	To what extent do you consider yourself ticklish?	single	all
**2**	Enjoyed child tickles?	Did you enjoy being tickled when you were a child?	single	all
**2**	Knismesis or gargalesis	There are two types of tickling: 1) itch-like sensation caused by light feather touch; 2) laughter-inducing vigorous touch on particular body parts including the armpits and soles of the feet. Which type of tickling do you prefer?	single	all
**3**	Tickling tool	Which of the following do you use for tickling? (Select all that apply)	multiple + free text	those who like tickling / being tickled
**3**	Tickling partner	Who do you tickle / who tickles you? (Select all that apply)	multiple + free text	those who like tickling / being tickled
**3**	Tickler or ticklee	Do you prefer to tickle or to be tickled?	single	those who like tickling / being tickled
**4**	Ticklers enjoy being tickled?	Do you enjoy being tickled?	single	those who like tickling (ticklers)
**4**	Body parts you enjoy tickling?	Please indicate which body parts of other people you prefer for each type of tickling and fetishism other than tickling (select all that apply)	multiple	those who like tickling (ticklers)
**4**		If you select "Other" body parts, please specify	free text	those who like tickling (ticklers)
**4**	Is tickling a sexual activity?	Do you consider tickling to be a sexual activity for you?	single	those who like tickling (ticklers)
**5**	Ticklees enjoy tickling?	Do you enjoy tickling someone?	single	those who like being tickled (ticklees)

**5**	Ticklishness change over time?	When you are tickled, does your level of ticklishness change over the course of a continuous tickling?	single	those who like being tickled (ticklees)
**5**	Body parts you enjoy being tickled?	Please indicate which body parts of yourself you prefer for being tickled with each type of tickling, and fetishism other than tickling (select all that apply)	multiple	those who like being tickled (ticklees)
**5**		If you select "Other" body parts, please specify	free text	those who like being tickled (ticklees)
**5**	Is tickling a sexual activity?	Do you consider tickling to be a sexual activity for you?	single	those who like being tickled (ticklees)
**6**	Ticklishness change over time?	When you are tickled, does your level of ticklishness change over the course of a continuous tickling?	single	those who prefer both to tickle and to be tickled equally
**6**	Body parts you enjoy tickling?	Please indicate which body parts of other people you prefer for each type of tickling and fetishism other than tickling (select all that apply)	multiple	those who prefer both to tickle and to be tickled equally
**6**		If you select "Other" body parts, please specify	free text	those who prefer both to tickle and to be tickled equally
**6**	Body parts you enjoy being tickled?	Please indicate which body parts of yourself you prefer for being tickled with each type of tickling, and fetishism other than tickling (select all that apply)	multiple	those who prefer both to tickle and to be tickled equally
**6**		If you select "Other" body parts, please specify	free text	those who prefer both to tickle and to be tickled equally
**6**	Is tickling a sexual activity?	Do you consider tickling to be a sexual activity for you?	single	those who prefer both to tickle and to be tickled equally
**7**	Aspect of arousal?	In what aspects of tickling someone do you experience sexual arousal? (Select all that apply)	multiple + free text	those who prefer to tickle someone as a sexual activity (sexual tickler)
**8**	Aspect of arousal?	In what aspects of being tickled do you experience sexual arousal? (Select all that apply)	multiple + free text	those who prefer to be tickled as a sexual activity (sexual ticklee)
**8**	Orgasm by tickling alone?	Do you experience orgasm (ejaculation in men) with tickling alone without genital stimulation?	single	those who prefer to be tickled as a sexual activity (sexual ticklee)
**8**	Tickle distress?	Do you experience distress when you are tickled?	single	those who prefer to be

				tickled as a sexual activity (sexual ticklee)
**9**	Aspect of arousal?	In what aspects of tickling someone do you experience sexual arousal? (Select all that apply)	multiple + free text	those who prefer both to tickle and to be tickled as a sexual activity (sexual tickler / ticklee)
**9**	Aspect of arousal?	In what aspects of being tickled do you experience sexual arousal? (Select all that apply)	multiple + free text	those who prefer both to tickle and to be tickled as a sexual activity (sexual tickler / ticklee)
**9**	Orgasm by tickling alone?	Do you experience orgasm (ejaculation in men) with tickling alone without genital stimulation?	single	those who prefer both to tickle and to be tickled as a sexual activity (sexual tickler / ticklee)
**9**	Tickle distress?	Do you experience distress when you are tickled?	single	those who prefer both to tickle and to be tickled as a sexual activity (sexual tickler / ticklee)
**10**	BDSM arousal?	Do you feel sexually aroused by BDSM acts other than tickling?	single	those who consider tickling is a sexual activity
**10**	Sexual satisfaction by tickling alone?	Is your sexual desire satisfied simply by tickling / being tickled?	single	those who consider tickling is a sexual activity
**10**	Sexual satisfaction without tickling?	To what extent can your sexual desire be satisfied by sexual activity that does not involve tickling?	single	those who consider tickling is a sexual activity
**10**	Post orgasm tickling desire decrease?	Do you experience a decrease in desire to tickle or be tickled after orgasm (ejaculation in men)?	single	those who consider tickling is a sexual activity
**10**	Tickling duration	How frequently do you engage in tickling as sexual activity?	single	those who consider tickling is a sexual activity
**10**	Tickling frequency	During a tickling session as a sexual activity, how long do you typically perform the tickling act continuously each time?	single	those who consider tickling is a sexual activity
**10**	Age of becoming sexually active	At what age did you first engage in sexual activity?	single	those who consider

				tickling is a sexual activity
**10**	Unwanted sex?	Have you ever experienced any unwanted sexual experiences during your childhood?	single	those who consider tickling is a sexual activity
**10**	Episode that led to tickle fetishism	Please describe any episode that you think led you to develop a tickling fetishism	free text	those who consider tickling is a sexual activity

Table S2: Gender-based statistics

	Males		Females				
**Ticklishness extent**	**N**	**%**	**N**	**%**	**Chi-square**	**DF**	***p*-value**
Completely unticklish	19	4%	0	0%			
Somewhat unticklish	51	10%	10	7%			
Neither ticklish nor unticklish	67	13%	9	6%			
Somewhat ticklish	221	41%	58	39%	18.53	4	< 0.001
Extremely ticklish	176	33%	72	48%			
**Total**	**534**	**100%**	**149**	**100%**			
**Tickle sensation preference**	**N**	**%**	**N**	**%**	**Chi-square**	**DF**	***p*-value**
I do like both equally	226	42%	72	48%			
Laughter-inducing vigorous touch	214	40%	33	22%			
Light feather touch	78	15%	42	28%	24.7531	3	< 0.001
I do not like either	16	3%	2	1%			
**Total**	**534**	**100%**	**149**	**100%**			
**To tickle or be tickled**	**N**	**%**	**N**	**%**	**Chi-square**	**DF**	***p*-value**
Both equally	198	38%	26	18%			
To tickle	194	37%	10	7%	133.5785	2	< 0.001
To be tickled	126	24%	111	76%			
**Total**	**518**	**100%**	**147**	**100%**			
**Enjoy childhood tickles**	**N**	**%**	**N**	**%**	**Chi-square**	**DF**	***p*-value**
Yes	244	46%	91	61%			
No	184	34%	32	21%	12.1728	2	0.002274
I am not sure / I don't remember	106	20%	26	17%			
**Total**	**534**	**100%**	**149**	**100%**			
**Ticklishness change**	**N**	**%**	**N**	**%**	**Chi-square**	**DF**	***p*-value**
It's getting somewhat less ticklish	60	19%	12	9%			
It's getting less and less ticklish	6	2%	4	3%			
Ticklishness does not change	44	14%	14	10%			
It's getting somewhat more ticklish	55	17%	38	28%	20.483	6	0.00227
It's getting more and more ticklish	59	18%	36	26%			
It's different every time	51	16%	23	17%			
I don't know	49	15%	10	7%			
**Total**	**324**	**100%**	**137**	**100%**			
**Ticklers enjoy being tickled**	**N**	**%**	**N**	**%**	**Chi-square**	**DF**	***p*-value**
I dislike being tickled	28	14%	1	10%			
I don't really like being tickled	39	20%	1	10%			
I'm neutral on being tickled	50	26%	4	40%	3.357	4	0.5
Being tickled is okay with me	51	26%	4	40%			

I enjoy being tickled	26	13%	0	0%			
**Total**	**194**	**100%**	**10**	**100%**			
**Ticklees enjoy tickling**	**N**	**%**	**N**	**%**	**Chi-square**	**DF**	***p*-value**
I dislike tickling someone	11	9%	5	5%			
I don't really enjoy tickling someone	10	8%	14	13%			
I'm neutral on tickling someone	22	17%	30	27%			
I somewhat enjoy tickling someone	44	35%	40	36%			
I enjoy tickling someone	39	31%	22	20%			
**Total**	**126**	**100%**	**111**	**100%**			
**Sexual arousal for being tickled**	**N**	**%**	**N**	**%**	**Chi-square**	**DF**	***p*-value**
Physical sensation	218	67%	73	53%			
Helplessness and submissiveness	174	54%	64	47%			
Anticipation	176	54%	58	42%	3.23	3	0.3575
Feeling enjoyable	0	0%	1	1%			
**Total**	**324**	**100%**	**137**	**100%**			
**Sexual arousal for tickling**	**N**	**%**	**N**	**%**	**Chi-square**	**DF**	***p*-value**
Ticklee's body reactions	285	73%	21	58%			
Ticklee's voice	271	69%	18	50%			
Sense of power	275	70%	17	47%			
Touching ticklee's body	217	55%	13	36%	0.621	6	0.996
Helplessness	1	0%	0	0%			
Partner's submission	1	0%	0	0%			
Tickle anticipation	1	0%	0	0%			
**Total**	**392**	**100%**	**36**	**100%**			

Table S3: Correlation matrix significance

		**Correlation coefficient**	***p*-value**	**DF**
BDSM arousal	sexual satisfaction without tickling	0.27	7.94E-06	847
current age	sexually active age	0.22	0.000	1092
sexual satisfaction by tickling	sexual satisfaction without tickling	-0.21	0.001	915
tickling frequency	sexual satisfaction without tickling	-0.20	0.001	795
sexual satisfaction by tickling	orgasm by tickling	0.24	0.003	793
tickling duration	post orgasm tickling desire decrease	-0.17	0.004	997
ticklishness extent	BDSM arousal	0.17	0.005	1164
ticklishness change	orgasm by tickling	0.22	0.006	628
orgasm by tickling	sexual satisfaction without tickling	-0.22	0.006	680
ticklishness extent	ticklers enjoy being tickled	0.25	0.007	926
ticklers enjoy being tickled	BDSM arousal	0.23	0.015	654
ticklers enjoy being tickled	current age	-0.22	0.020	926
ticklishness extent	tickling duration	0.14	0.024	1232
ticklishness change	sexually active age	-0.17	0.031	723
ticklishness change	tickling frequency	0.17	0.032	743
ticklishness extent	current age	-0.13	0.036	1436
tickling frequency	tickling duration	0.13	0.038	908
ticklers enjoy being tickled	sexually active age	-0.19	0.041	582
tickling frequency	sexual satisfaction by tickling	0.12	0.054	908
ticklishness extent	post orgasm tickling desire decrease	-0.12	0.059	1201
tickling duration	tickle distress	0.15	0.067	853
ticklers enjoy being tickled	sexual satisfaction without tickling	0.17	0.072	609
ticklishness change	ticklees enjoy tickling	0.21	0.073	599
current age	ticklees enjoy tickling	-0.21	0.078	968
tickling duration	ticklees enjoy tickling	-0.20	0.094	764
tickling duration	current age	0.10	0.100	1232
sexual satisfaction by tickling	post orgasm tickling desire decrease	-0.10	0.113	997
tickling frequency	orgasm by tickling	0.13	0.113	673
tickling duration	sexual satisfaction without tickling	-0.10	0.117	915
ticklishness change	sexual satisfaction without tickling	-0.12	0.127	750
ticklishness change	post orgasm tickling desire decrease	-0.12	0.130	832
ticklishness extent	ticklishness change	0.12	0.134	1067
ticklishness change	sexual satisfaction by tickling	0.12	0.138	863
orgasm by tickling	post orgasm tickling desire decrease	-0.12	0.145	762
tickling frequency	tickle distress	0.12	0.153	733
tickle distress	sexually active age	0.11	0.156	713
sexually active age	ticklees enjoy tickling	-0.16	0.171	624
orgasm by tickling	sexually active age	-0.11	0.190	653
BDSM arousal	tickling duration	0.08	0.202	960
BDSM arousal	sexually active age	-0.07	0.232	820
tickle distress	current age	-0.09	0.243	1057

sexual satisfaction by tickling	tickling duration	0.06	0.293	1028
sexual satisfaction by tickling	sexually active age	0.06	0.302	888
ticklers enjoy being tickled	tickling frequency	0.10	0.311	602
orgasm by tickling	current age	-0.08	0.336	997
tickling frequency	post orgasm tickling desire decrease	-0.06	0.342	877
BDSM arousal	orgasm by tickling	-0.08	0.352	725
ticklishness extent	sexually active age	-0.06	0.352	1092
ticklishness extent	ticklees enjoy tickling	0.11	0.354	968
ticklishness change	tickling duration	0.07	0.375	863
tickle distress	post orgasm tickling desire decrease	-0.07	0.383	822
tickle distress	ticklees enjoy tickling	-0.10	0.393	589
sexual satisfaction by tickling	ticklees enjoy tickling	0.10	0.403	764
sexually active age	sexual satisfaction without tickling	-0.05	0.408	775
sexual satisfaction without tickling	post orgasm tickling desire decrease	0.05	0.412	884
ticklishness extent	sexual satisfaction without tickling	0.05	0.430	1119
ticklees enjoy tickling	post orgasm tickling desire decrease	-0.09	0.466	733
BDSM arousal	tickling frequency	-0.04	0.467	840
ticklishness extent	orgasm by tickling	0.05	0.519	997
ticklees enjoy tickling	sexual satisfaction without tickling	-0.08	0.519	651
sexually active age	post orgasm tickling desire decrease	0.04	0.533	857
ticklers enjoy being tickled	tickling duration	0.06	0.554	722
BDSM arousal	sexual satisfaction by tickling	-0.03	0.579	960
tickling frequency	current age	0.03	0.588	1112
BDSM arousal	current age	0.03	0.615	1164
tickle distress	orgasm by tickling	-0.04	0.627	618
ticklers enjoy being tickled	post orgasm tickling desire decrease	-0.05	0.633	691
ticklishness change	current age	-0.04	0.642	1067
BDSM arousal	post orgasm tickling desire decrease	-0.02	0.701	929
ticklishness extent	tickle distress	0.03	0.737	1057
current age	post orgasm tickling desire decrease	0.02	0.749	1201
ticklers enjoy being tickled	sexual satisfaction by tickling	0.03	0.769	722
BDSM arousal	ticklishness change	0.02	0.783	795
tickle distress	sexual satisfaction without tickling	-0.02	0.823	740
sexual satisfaction by tickling	tickle distress	0.02	0.829	853
tickling frequency	ticklees enjoy tickling	0.03	0.832	644
ticklishness extent	sexual satisfaction by tickling	-0.01	0.849	1232
current age	sexual satisfaction without tickling	0.01	0.851	1119
BDSM arousal	tickle distress	-0.01	0.856	785
tickling duration	sexually active age	0.01	0.867	888
ticklishness change	tickle distress	-0.01	0.892	688
tickling frequency	sexually active age	0.01	0.910	768
ticklishness extent	tickling frequency	0.00	0.937	1112
BDSM arousal	ticklees enjoy tickling	-0.01	0.961	696
tickling duration	orgasm by tickling	0.00	0.964	793

orgasm by tickling	ticklees enjoy tickling	0.00	0.987	529
sexual satisfaction by tickling	current age	0.00	0.997	1232

Table S4: Exploratory factor analysis of tickle fetishism

**Variables**	**Factor 1**	**Factor 2**	**Factor 3**	**Communality**	**Uniqueness**
Sexual satisfaction without tickling	-0.5	0.3	-0.1	0.4	0.6
Sexual satisfaction by tickling	0.4	0.1	0.1	0.2	0.8
Orgasm by tickling	0.4	0.0	-0.2	0.2	0.8
Tickling frequency	0.4	0.0	0.1	0.2	0.8
Ticklishness change	0.4	0.2	-0.2	0.2	0.8
Ticklishness extent	0.0	0.5	-0.1	0.2	0.8
BDSM arousal	-0.2	0.5	0.1	0.3	0.7
Post orgasm tickling desire decrease	-0.1	-0.3	0.0	0.1	0.9
Sexually active age	0.0	-0.1	0.5	0.3	0.7
Current age	0.0	0.0	0.4	0.1	0.9
Tickling duration	0.3	0.2	0.3	0.2	0.8
Tickle distress	0.1	0.1	0.2	0.0	1.0
